# No change in reoperation rates despite shifting treatment trends: a population-based study of 4,070 proximal humeral fractures

**DOI:** 10.1080/17453674.2021.1941629

**Published:** 2021-06-30

**Authors:** Carl Bergdahl, David Wennergren, Eleonora Swensson-Backelin, Jan Ekelund, Michael Möller

**Affiliations:** a Department of Orthopaedics, Institute of Clinical Sciences, Sahlgrenska Academy, University of Gothenburg, Gothenburg; b Department of Orthopaedics, Sahlgrenska University Hospital, Gothenburg/Mölndal; c Centre of Registers, Western Healthcare Region, Gothenburg, Sweden

## Abstract

Background and purpose — Clear and acknowledged treatment algorithms for proximal humeral fractures (PHFs) are lacking. Nevertheless, a change in treatment trends, including a change towards more reversed shoulder arthroplasties (RSA), has been observed during recent years. We examined the effect of these changes on reoperation rates.

Patients and methods — Between 2011 and 2017, 4,070 PHFs treated at Sahlgrenska University Hospital were registered prospectively in the Swedish Fracture Register (SFR) and followed up until 2019 (mean follow-up of 4.5 years). Data on all reoperations were gathered from the SFR and from medical records.

Results — The majority of PHFs were treated non-surgically and the proportion increased slightly, but not statistically significantly, during the study period (from 76% to 79%). Of the surgically treated fractures, the proportion fixed with a plate decreased from 47% to 25%, while the use of RSA increased 9-fold (from 2.0% to 19%). 221 patients underwent 302 reoperations. For those primarily treated surgically, the reoperation rate was 17%. Among treatment modalities, plate fixation was associated with the highest reoperation rate (21%). Rate of reoperations remained constant during the study period, both for the entire study cohort and for the surgically treated patients

Interpretation — During the study period, treatment changes that are in accordance with recently published treatment recommendations were observed. However, these treatment changes did not affect the reoperation rate. Treatment with a plate, intramedullary nail, or hemiarthroplasty was associated with the highest reoperation rates. The fact that almost every 4th surgical procedure was a reoperation indicates a need for further improvement of modern treatment concepts for PHFs.

Fractures of the proximal humerus are common and are associated with a long-term negative impact on quality of life and excess mortality (Clement et al. 2014, Bergdahl et al. 2020). The optimal treatment is controversial. Displaced proximal humeral fractures (PHF) may result in poor outcome regardless of treatment modality and challenging revision procedures are common (Olerud et al. 2011a, 2011b, Lange et al. 2016).

The treatment options for PHFs have evolved rapidly in recent years. The introduction of locking plates at the beginning of this century led to a sharp increase in the surgical fixation of PHFs (Bell et al. 2011, Sumrein et al. 2017). However, this trend was accompanied by an increased rate of complications and reoperations (Bell et al. 2011). In attempts to reduce the failure and revision rate, multiple modified surgical techniques evolved (Barlow et al. 2011, Boileau et al. 2013). Parallel to this development, reversed shoulder arthroplasties (RSAs) became increasingly popular (Han et al. 2016). Compared with the traditional fixation methods, RSA demonstrated lower complication rates (Klug et al. 2019). As a result, RSA use has increased markedly, while plate fixation has decreased (Han et al. 2016, Rajaee et al. 2017).

According to the latest Cochrane review on PHF interventions there is a lack of data from randomized controlled trials (RCTs) to support one treatment over another (Handoll and Brorson 2015). The complication rate and need for revision surgery are therefore relevant measurements when evaluating PHF treatment. It is important to examine whether changes in treatment affect the need for repeat surgery. We evaluated trends in treatment methods for PHFs at a large Swedish orthopedic trauma unit and explored the rate of and risk factors for reoperations after primary treatment.

## Patients and methods

### Study population

All patients aged ≥ 16 years treated for a PHF at Sahlgrenska University Hospital (SUH) in Gothenburg in 2011–2017 were identified in the Swedish Fracture Register (SFR). Patients were followed until December 31, 2019, with an average follow-up time of 4.5 years (2–9).

Data on patient demographics (age, sex), fracture characteristics according to the AO/OTA (Arbeitsgemeinschaft für Osteosynthesefragen/Orthopaedic Trauma Association) classification and treatment (primary treatment, cause, and type of late surgery/reoperation) were extracted from the SFR. By using the Swedish Tax Agency population register, all deaths during follow-up were identified. The implementation, design, validation, and registration process of the SFR have been described previously (Wennergren et al. 2015). SUH is the sole provider of orthopedic trauma care in Gothenburg and is also responsible for all sequalae related to fractures. To ensure that all reoperations were included, the digital surgical planning system at SUH was checked for all patients included in the study. Medical records were reviewed in search of absent registrations in the SFR and missed treatments were included in this study. The reporting of this observational study follows the STROBE guidelines.

### Treatment

Indication for treatment was not standardized, but all PHFs were managed by a small group of experienced orthopedic trauma surgeons specialized in upper extremity trauma. Surgical management was prompted for severely displaced fractures in patients with high functional demands of their shoulder or when deemed compulsory. “Primary treatment” was defined as the treatment given within 30 days of the fracture date, as treatment can be altered for a PHF at an early (≤ 14 days) follow-up visit. As a result, nonoperatively treated patients who subsequently (within 30 days) underwent delayed surgery were classified as primarily surgically treated. Those treated non-surgically were immobilized in a sling for 2–4 weeks followed by physiotherapy.

Surgical treatment was divided into 5 groups: locking plate, intramedullary nail (IM nail), hemi-arthroplasty (HA), reverse shoulder arthroplasty (RSA), and a combination of methods (combination). The combination methods included fixation with screws, cerclage wires, suture anchors, mini-plates, or a combination thereof typically used for displaced tuberosity fractures. Both cemented and uncemented HAs and RSAs were included. Trends in treatment were evaluated, and comparisons were made between the beginning (2011–2012) and the end of the study period (2016-2017).

### Reoperations

All surgical interventions following an initial surgical treatment were referred to as a “reoperation.” In addition, a late surgical procedure (≥ 30 days) following an initial non-surgical treatment was regarded as a “reoperation.” Reasons for reoperations were analyzed (non-union, malunion, avascular necrosis [AVN] with collapse [Cruess grade 4 or 5], infection, implant failure, and reoperation due to patient demands). Secondary displacements with screw penetration, perioperative misplacement of implants, instability, and tuberosity absorption/displacement/malfunction were included in the “implant failure” group. Implant removal is not routinely performed at our institution and all reoperations were performed for symptomatic failures in agreement with the patient. In accordance with Olerud et al. (2011b), reoperations were further divided into major and minor reoperations. Major reoperations included all reoperations deemed compulsory, i.e., reoperations for all causes apart from reoperations due to patient demands.

### Statistics

Changes in patient demographics and treatment between the beginning (2011–2012) and the end (2016–2017) of the study period were analyzed using chi-square and Student’s t-test for categorical and continuous variables, respectively. Statistical significance was set at p-values of < 0.05. Kaplan–Meier survival analysis was undertaken to illustrate the cumulative survival rate (i.e., time to reoperation) for the different treatment modalities. Date of death was used as censor and reoperation as event in the analyses, while the end of follow-up was December 31, 2019. Risk factors for reoperations were analyzed with a Cox proportional hazards regression model and expressed as hazard ratios (HR) with 95% confidence intervals (CI). IBM SPSS statistics version 25.0 (IBM Corp, Armonk, NY, USA) was used for all statistical analyses.

### Ethics, funding, data sharing, and potential conflicts of interest

The study was conducted in accordance with the Helsinki Declaration and was approved by the Central Ethical Review Board in Gothenburg, Sweden (reference number T1137-18). In accordance with Swedish legislation, individual consent was not required. No grants from any public, commercial, or not-for-profit sector were received for this study. The data that supports the findings of this study is available from the corresponding author on reasonable request.

The authors declare no competing interests.

## Results

During the study period, 4,009 patients with PHFs were registered in the SFR at SUH. 22 patients were excluded due to primary treatment elsewhere (n = 7), follow-up at another hospital (n = 13), and excision arthroplasty (n = 2), leaving 3,987 patients with 4,070 PHFs for the final analysis. The mean age at the time of a PHF was 68 years (16–104) and 72% of the patients were female. According to the AO/OTA classification system, most fractures were classified as type A (49%), followed by type B (39%), and type C (11%). The majority were treated nonoperatively (77%), while 23% of PHFs were treated surgically (Table 1). Among surgical treatments, fixation with a locking plate was most common (35%), followed by fixation with an IM nail (27%). Most fractures treated with a combination method were of AO/OTA type A (81%), of which 101 fractures (94%) were isolated tuberosity fractures (AO/OTA 11-A1). Patients treated with an arthroplasty (HA and RSA) had the highest proportion of complex fractures (AO/OTA type C 76% and 66% respectively). No fractures were treated with percutaneous methods requiring mandatory removal of the fixation device.

**Table 1.** Frequency of treatment modalities with demographic data and distribution of fracture types for the study cohort

Treatment modality **^a^**	Number of fracturesn (%)	Age mean (range)	Female %	Fracture type (AO/OTA ^b^) n (%) **^c^**		
				A	B	C
Non-surgical	3,117 (77)	68 (16–104)	73	1,653 (53)	1,299 (42)	165 (5.3)
Surgical	953 (23)	65 (16–103)	68	347 (36)	307 (32)	299 (31)
Plate	332 (8.2)	60 (16–99)	66	58 (17)	170 (51)	104 (31)
IM nail	255 (6.3)	71 (19–103)	69	164 (64)	71 (28)	20 (7.8)
Combination method	134 (3.3)	57 (20–102)	54	108 (81)	17 (13)	9 (6.7)
HA	139 (3.4)	70 (35-92)	75	8 (5.8)	26 (19)	105 (76)
RSA	93 (2.3)	75 (51–96)	85	9 (9.7)	23 (25)	61 (66)
All treatments	4,070 (100)	68 (16–104)	72	2,000 (49)	1,606 (39)	464 (11)

**^a^**IM nail, intramedullary nail; HA, hemiarthroplasty; RSA, reverse shoulder arthroplasty.

**^b^**AO/OTA, Arbeitsgemeinschaft für Osteosynthesefragen/Orthopaedic Trauma Association fracture classification; A, fracture type A; B, fracture type B; C, fracture type C.

**^c^**Percentage within the treatment group.

The completeness in the SFR for primary procedures and reoperations was 97% (n = 928/953) and 62% (n = 188/302) respectively when medical charts were reviewed.

### Change in treatment practice

**Figure 1.**
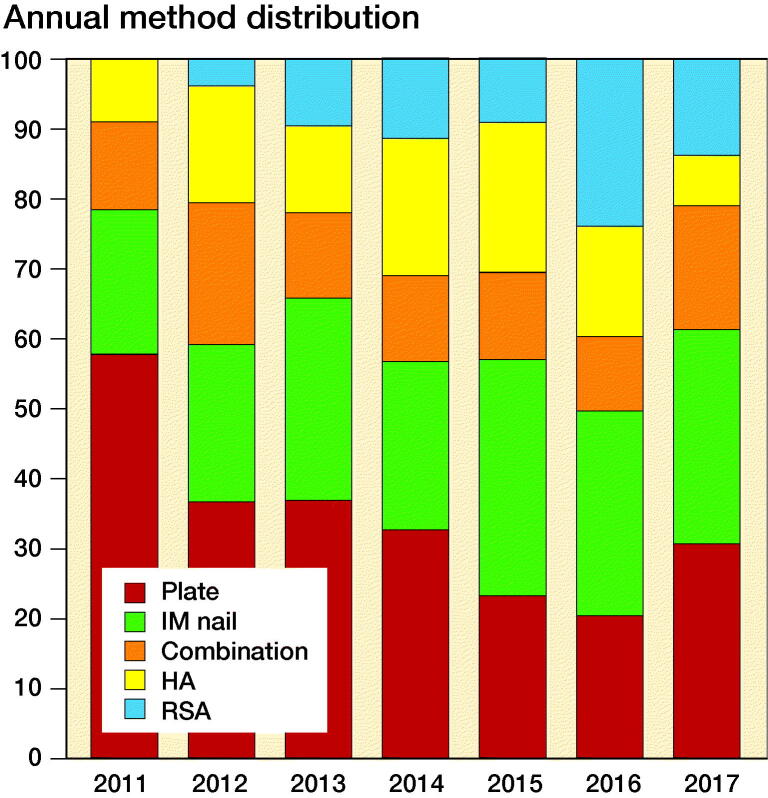
Trends in treatment of surgically treated proximal humeral fractures (PHFs), presented by treatment modality as the proportion (%) of the total number of surgically treated PHFs for each year during the study period.

Throughout the 7-year study period, there were no statistically significant differences regarding patient demographics (age and sex) and fracture characteristics (AO/OTA groups). The proportion of surgically treated fractures did not differ statistically significantly during the study period (24% in 2011–2012 vs. 21% in 2016–2017; p = 0.06), but the distribution among the treatment modalities within the surgical group did. The proportion treated with a plate almost halved (n = 138/293; 47% in 2011–2012 vs. n = 61/237; 26% in 2016–2017; p < 0.001; Figure 1), while the proportion treated with IM nails substantially increased (n = 63/293; 22% in 2011–2012 vs. n = 71/237; 30% in 2016–2017; p = 0.03). However, the greatest proportional increase was seen in the group treated with RSA (n = 6/293; 2.0% in 2011–2012 vs. n = 44/237; 19% in 2016–2017; p < 0.001).

### Reoperation/unplanned surgery

During the 7-year study period, 221 patients underwent 302 reoperations. 1 of almost every 4 surgical interventions (302/1,257; 24%) for a PHF during the study period was a reoperation. The overall reoperation rate (including late surgery following initial non-surgical treatment) was 5.4% regardless of cause and 4.0% when considering only major reoperations (Table 2). Among the surgically treated fractures, 118 (12%) were subjected to a major reoperation and 163 (17%) to a reoperation of any cause.

**Table 2.** Number, frequency, and reason for the first reoperation by treatment modality. Values are count (%)

Modality	Total no. of patients	Fractures reoperated		Nonunion	Malunion	Reason for first reoperation			Patient demands	Other **^b^**	Number of reoperations **^c^**
		All **^a^**	Major reop. **^a^**			AVN	Infection	Implant failure			
Non-surgical	3,117	58 (1.9)	46 (1.5)	27 (0.87)	12 (0.38)	6 (0.19)	1 (0.03)	–	12 (0.38)	–	78 (6.2)
Surgical	953	163 (17)	118 (12)	2 (0.21)	4 (0.42)	22 (2.3)	13 (1.4)	77 (8.1)	44 (4.6)	1 (0.10)	224 (18)
Plate	332	70 (21)	51 (15)	1 (0.30)	3 (0.90)	17 (5.1)	7 (2.1)	22 (6.6)	19 (5.7)	1 (0.30)	103 (8.2)
IM nail	255	46 (18)	32 (13)	–	–	5 (2.0)	–	27 (11)	14 (5.5)	–	51 (4.0)
Combination method	134	18 (13)	11 (8.2)	–	1 (0.75)	1 (0.75)	1 (0.75)	9 (6.7)	6 (4.5)	–	24 (1.9)
HA	139	23 (17)	19 (14)	–	–	–	3 (2.2)	16 (12)	4 (2.9)	–	32 (2.5)
RSA	93	6 (6.5)	5 (5.4)	–	–	–	2 (2.2)	3 (3.2)	1 (1.1)	–	14 (1.1)
All treatments	4,070	221 (5.4)	164 (4.0)	9 (0.71)	16 (0.39)	28 (0.69)	14 (0.34)	77 (1.9)	56 (1.3)	1 (0.002)	302 (24)

IM nail, intramedullary nail; HA = hemiarthroplasty; RSA, reverse shoulder arthroplasty; AVN, avascular necrosis.

**^a^**Percentage within treatment group.

**^b^**Reoperated with arthrodesis due to axillary nerve palsy sustained at initial trauma.

**^c^**Percentage within all surgical procedures.

Despite the observed changes in treatment practice during the study period, the reoperation frequency remained similar between the beginning of the study and the end (all PHFs all reoperations 5.5% in 2011–2012 vs. 5.1% 2016–2017, all PHFs major reoperations 3.8% in 2011–2012 vs. 4.2% in 2016–2017, surgically treated PHFs all reoperations 18% in 2011–2012 vs. 16% in 2016–2017, surgically treated PHFs major reoperations 13% in 2011–2012 vs. 13% in 2016–2017).

**Figure 2.**
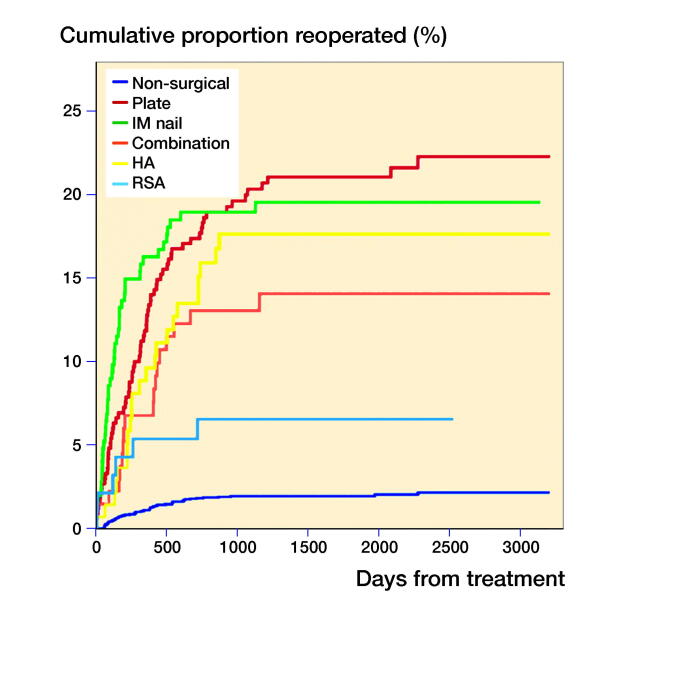
Kaplan–Meier curves, split by treatment modality, showing risk of reoperation over time.

The most common reason for the first reoperation was implant failure (35%) followed by reoperation on patient demands, most often due to impaired range of motion and/or pain (25%; Table 3). Reoperations due to infection were less common (6%). However, most infections required more than 1 surgical procedure, so infection was the underlying cause for 12% of all reoperations. Most reoperations occurred within 2 years of the initial treatment (Figure 2) and the treatment modality associated with the highest rate of reoperation was plate fixation (all cause reoperations 21% and major reoperations 15%). The majority of major reoperations following plate fixation were due to implant failure (43%) or AVN (33%). Among the surgical treatment modalities, patients treated with RSA had the lowest reoperation rate (all cause reoperations 6% and major reoperations 5%).

**Table 3.** Indication for reoperation: frequency, time to reoperation, and total number of reoperations

Indication for reoperation	Total number of reoperated fractures n (%)	Days from treatment to first reoperation mean (range)	Total number of reoperations n (%)
Nonunion	29 (13)	194 (59–658)	45 (15)
Malunion	16 (7.2)	651 (203–2,247)	20 (6.6)
AVN	28 (13)	487 (76–1,946)	40 (13)
Infection	14 (6.3)	171 (9–861)	37 (12)
Implant failure	77 (35)	187 (1–727)	99 (33)
Patient demands	56 (25)	500 (47–2,247)	60 (20)
Other **^a^**	1 (0.5)	158 (	1 (0.3)
Total	221 (100)	338 (1–2,247)	302 (100)

AVN, avascular necrosis.

**^a^** Reoperated with arthrodesis due to axillary nerve palsy sustained at initial trauma.

### Risk factors for reoperations

The risk of a major reoperation increased with fracture complexity (HR 1.6 [CI 1.1–2.4] in AO/OTA type B and HR 5.7 [CI 3.6–8.4] in type C compared with type A; Table 4). Younger age (< 59 years) was an independent predictor of reoperation when all reoperations were analyzed. However, when patient-requested reoperations were excluded from the analysis, the increased risk with younger age became less apparent (Table 4). Neither sex nor injury mechanism (high- or low-energy trauma) was associated with an increased risk of reoperation.

**Table 4.** Cox proportional hazards regression of independent predictors of reoperation following treatment for a proximal humeral fracture

	All reoperations			Major reoperations		
Risk factor	p-value	Exp(B)	(95%CI)	p-value	Exp(B)	(95% CI)
Age (≤ 59 years as reference)						
60–74	0.4	0.86	(0.63–1.2)	0.5	1.2	(0.78–1.7)
75–84	< 0.001	0.37	(0.23–0.59)	0.04	0.57	(0.34–0.96)
≥ 85	0.007	0.50	(0.30–0.83)	0.5	0.84	(0.49–1.5)
Sex (male as reference)						
Female	0.8	1.0	(0.93–1.8)	0.8	0.95	(0.67–1.4)
AO/OTA group (Group A as reference)						
Group B	0.1	1.3	(0.93–1.8)	0.02	1.6	(1.1–2.4)
Group C	< 0.001	4.9	(3.5–6.8)	0.001	5.7	(3.8–8.4)
Injury mechanism **^a^** (high-energy trauma as reference)						
Low-energy trauma	0.2	0.71	(0.43–1.2)	0.3	0.71	(0.38–1.3)

AO/OTA group, see Table 1

**^a^** 87 fractures excluded due to unknown (71) or inapplicable (16) trauma mechanism.

## Discussion

This large prospectively collected but retrospectively validated and reviewed cohort study of PHF treatment in Gothenburg demonstrates that the proportion of patients treated surgically decreased slightly (although statistically non-significantly, p = 0.06) from 2011 to 2017, and that there was a significant change in surgical treatment modalities. The use of plate fixation decreased in favor of IM nails and RSAs. However, these changes in treatment have had no effect on the reoperation frequency. The reoperation frequency remained constant, 5% for all patients, 2% for nonoperatively treated patients, and 17% for surgically treated patients. Independent risk factors for reoperations were fracture complexity (AO/OTA type C) and younger age (< 59 years).

PHFs are most commonly managed by non-surgical treatment. In our study, the proportion of non-surgical treatment was 76% in 2011/2012 and 79% in 2016/2017. After a decade of increasing rates of surgical treatment, a plateau appears to have been reached regarding surgical/non-surgical treatment. 2 studies from the United States reported a relative increase of 26% and 56% respectively of surgically managed PHFs from the late 1990s to 2005 (Bell et al. 2011; Petrigliano et al. 2014). A similar trend was reported in Sweden, with a doubling of the relative rate of surgical treatment for PHFs in 2000–2012 (Sumrein et al. 2017). The plateauing trend we observed might be the result of the numerous publications from 2010 and onwards, reporting a non-superior patient-reported outcome following surgical treatment compared with non-surgical treatment for PHFs (Olerud et al. 2011a, 2011b, Rangan et al. 2015, Launonen et al. 2019).

The decreased use of plate fixation and the increased use of IM nails and RSAs in this study are in accordance with previous reports from the western world. Both Rajaea et al. (2017) and Rosas et al. (2016) reported a decrease in the rate of plate fixation and a doubling in the rate of RSA use for PHFs in the US between 2011–2013 and 2009–2012 respectively. Several other studies have demonstrated the increasing role of RSA in the treatment of PHFs, especially in older patients with complex fractures. Registry data from the Nordic countries and New Zealand demonstrated a 5- to 6-fold increase in the incidence of RSA for PHFs between 2009 and 2016 (van der Merwe et al. 2017, Lehtimäki et al. 2020). Parallel to the growing popularity of RSA in recent years, an increased role for IM nails has been recognized, especially among elderly patients. An RCT from 2011 demonstrated lower complication rates associated with the modern version of IM nails compared with locking plates for two-part surgical-neck fractures (AO/OTA A2 and A3) (Zhu et al. 2011).

Contrary to what we anticipated, the treatment changes we found did not render lower overall reoperation rates. One possible explanation might be the observation that reoperations following fixation with a plate, IM nail, or HAs remained high during the study period. With recent treatment changes, including advances in surgical techniques and the preferred use of RSA instead of plate fixation in the elderly, as well as in complex fractures, a reduced failure rate/reoperation rate for these treatment modalities could be expected. However, no such changes were noted. Because plate fixation, IM nails, and HA still accounted for two-thirds (n = 159, 67%) of all surgical treatments at the end of the study period, the low reoperation rates following RSA did not result in lower overall reoperation rates.

Considering the high rate of reported reoperations following plate, IM nail fixation, and HAs, these treatments must be questioned, especially as their superiority compared with nonoperative treatment has not been demonstrated (Olerud et al. 2011a, 2011b, Lange et al. 2016, Launonen et al. 2019). In fact, no surgical treatment modality has demonstrated superior functional and/or patient-reported outcome compared with non-surgical treatment for displaced PHFs, but surgery has been associated with substantially higher risks of secondary surgery with a risk ratio of 2.2 (95% CI 1.2–4.0) at 2 years post-fracture (Handoll and Brorson 2015, Lopiz et al. 2019). The fact that almost a quarter of all surgical procedures for PHFs in this study were unplanned reoperative procedures implies a need for additional research on how to select patients and fractures for the respective treatment in order to minimize the need for reoperations.

RSA was associated with the lowest reoperation rates of all surgical treatment modalities in our study. However, this should be interpreted with caution because revision surgery following RSA is particularly demanding and few salvage procedures are available. Nevertheless, a recent Swedish study demonstrated superior clinical results in patients treated with RSA compared with HA and, taken together with the low revisions rates, these results indicate that RSA plays an important role in the surgical management of PHFs (Jonsson et al. 2021).

Few previous studies have reported overall reoperation rates in a consecutive series of PHFs, regardless of treatment modality. We found no other study with such a large cohort of patients that had been individually controlled for reoperations. In a Swiss study, 192 consecutive patients with a PHF were followed up for a year and the reoperation rate of 11% was higher than the 5% reported in our study (Spross et al. 2019). In our study, reoperation was used as an indication of a complication or failure of the primary treatment. However, repeat surgery as an outcome measurement must always be interpreted with caution. The absence of reoperation does not necessarily represent an acceptable outcome. Many patients with complications or poor functional outcome are not subjected to reoperation (Amundsen et al. 2019, Barlow et al. 2020). Therefore the actual rate of complication or poor functional outcome in our study was probably substantially higher than the reported reoperation rates.

Younger age and increasing fracture complexity were found to be independent predictors of reoperation, which are in accordance with previous reports (Petrigliano et al. 2014, Barlow et al. 2020). Younger patients are probably more willing to undergo further surgery in order to avoid dysfunction and pain, which may be an explanation for the increased risk. This assumption is supported by the higher rate of patient-requested reoperations we found in patients ≤ 59 years old. Similar findings have been demonstrated for other surgically treated fractures (Wennergren et al. 2021). Elderly patients are generally reluctant to undergo additional surgery and probably more inclined to accept dysfunction. Barlow et al. (2020) found that older age was associated with increased radiological complications in a study of plate-fixated PHFs. Nevertheless, older patients were less likely to undergo reoperations.

High validity for reoperations is a proven difficulty in register-based studies and underreporting leads to an underestimation of the risk of reoperation (Wennergren et al. 2021). To avoid reoperations not registered in the SFR remaining undetected, only patients primarily treated and eligible for follow-up at SUH were included. This enabled a medical-chart review in the search for reoperations. Reoperations performed outside SUH would consequently not be included. However, those are most likely few in numbers considering that SUH is the sole provider of treatment for PHFs and sequalae related to PHFs in the region.

### Strengths and limitations

There are some limitations to this study. The data were collected prospectively but reviewed retrospectively. Thus, as previously mentioned, the risk of reoperations not being included in the study cannot be fully disregarded. On the other hand, the study design enabled the inclusion of more than 4,000 consecutive and individually reviewed PHFs, which is a considerable strength. This adds important knowledge to the evaluation of the overall treatment for PHFs outside the strict setting of RCTs. Another limitation always to be considered in research on PHFs is the limited interobserver reliability reported for PHF classifications (Wennergren et al. 2017). Lastly, the results are based on data from a single center, which may limit the generalizability. This was, however, a prerequisite in order to obtain a high level of completeness and validity regarding reoperations.

## Conclusion

This study provides an evaluation of recent trends in PHF management with regards to reoperations. Although RSAs and IM nailing increased substantially, while plate fixation decreased, no effect was observed in relation to reoperation rates. Plate fixation, IM nailing, and HAs continue to be associated with high failure rates and 1 in every 4 surgical interventions for a PHF was a reoperation. These results highlight the need for a better treatment algorithm to optimize the care of patients with PHFs.

CB and MM conceived the study idea and ESB and CB conducted the completion of data. CB and JE performed the statistical analyses and CB wrote the initial draft. All authors contributed to the interpretation of the data and revision of the manuscript.

The authors wish to thank all the orthopedic surgeons at the affiliated department for entering detailed data on busy working days. 

*Acta* thanks Stig Brorson and Antti P Launonen for help with peer review of this study.

## References

[CIT0001] Amundsen A, Rasmussen J V, Olsen B S, Brorson S. Low revision rate despite poor functional outcome after stemmed hemiarthroplasty for acute proximal humeral fractures: 2,750 cases reported to the Danish Shoulder Arthroplasty Registry. Acta Orthop 2019; 90(3): 196–201. 30931676 10.1080/17453674.2019.1597491 PMC6534238

[CIT0002] Barlow J D, Sanchez-Sotelo J, Torchia M. Proximal humerus fractures in the elderly can be reliably fixed with a “hybrid” locked-plating technique. Clin Orthop Relat Res 2011; 469: 3281–91. 21479762 10.1007/s11999-011-1894-y PMC3210261

[CIT0003] Barlow J D, Logli A L, Steinmann S P, Sems S A, Cross W W, Yuan B J, Torchia M E, Sanchez-Sotelo J. Locking plate fixation of proximal humerus fractures in patients older than 60 years continues to be associated with a high complication rate. J Shoulder Elbow Surg 2020; 29: 1689–94. 32088075 10.1016/j.jse.2019.11.026

[CIT0004] Bell J E, Leung B C, Spratt K F, Koval K J, Weinstein J D, Goodman D C, Tosteson A N. Trends and variation in incidence, surgical treatment, and repeat surgery of proximal humeral fractures in the elderly. J Bone Joint Surg Am 2011; 93: 121–31. 21248210 10.2106/JBJS.I.01505 PMC3016042

[CIT0005] Bergdahl C, Wennergren D, Ekelund J, Möller M. Mortality after a proximal humeral fracture. Bone Joint J 2020; 102-b: 1484–90. 33135440 10.1302/0301-620X.102B11.BJJ-2020-0627.R1

[CIT0006] Boileau P, Winter M, Cikes A, Han Y, Carles M, Walch G, Schwartz D G. Can surgeons predict what makes a good hemiarthroplasty for fracture? J Shoulder Elbow Surg 2013; 22: 1495–506. 23834993 10.1016/j.jse.2013.04.018

[CIT0007] Clement N D, Duckworth A D, McQueen M M, Court-Brown C M. The outcome of proximal humeral fractures in the elderly: predictors of mortality and function. Bone Joint J 2014; 96-b: 970–7. 24986953 10.1302/0301-620X.96B7.32894

[CIT0008] Han R J, Sing D C, Feeley B T, Ma C B, Zhang A L. Proximal humerus fragility fractures: recent trends in nonoperative and operative treatment in the Medicare population. J Shoulder Elbow Surg 2016; 25: 256–61. 26440695 10.1016/j.jse.2015.07.015

[CIT0009] Handoll H H, Brorson S. Interventions for treating proximal humeral fractures in adults. Cochrane Database Syst Rev 2015; CD000434. 26560014 10.1002/14651858.CD000434.pub4

[CIT0010] Jonsson E Ö, Ekholm C, Salomonsson B, Demir Y, Olerud P. Reverse total shoulder arthroplasty provides better shoulder function than hemiarthroplasty for displaced 3- and 4-part proximal humeral fractures in patients over 70 years of age: a multicenter randomized controlled trial. J Shoulder Elbow Surg 2021; 30(5): 994–1006. 33301926 10.1016/j.jse.2020.10.037

[CIT0011] Klug A, Wincheringer D, Harth J, Schmidt-Horlohé K, Hoffmann R, Gramlich Y. Complications after surgical treatment of proximal humerus fractures in the elderly: an analysis of complication patterns and risk factors for reverse shoulder arthroplasty and angular-stable plating. J Shoulder Elbow Surg 2019; 28: 1674–84. 31056394 10.1016/j.jse.2019.02.017

[CIT0012] Lange M, Brandt D, Mittlmeier T, Gradl G. Proximal humeral fractures: non-operative treatment versus intramedullary nailing in 2-, 3- and 4-part fractures. Injury 2016; 47 Suppl 7: S14–s19. 28040071 10.1016/S0020-1383(16)30848-8

[CIT0013] Launonen A P, Sumrein B O, Reito A, Lepola V, Paloneva J, Jonsson K B, Wolf O, Ström P, Berg H E, Felländer-Tsai L, Jansson K Å, Fell D, Mechlenburg I, Døssing K, Østergaard H, Märtson A, Laitinen M K, Mattila V M. Operative versus non-operative treatment for 2-part proximal humerus fracture: a multicenter randomized controlled trial. PLoS Med 2019; 16: e1002855. 31318863 10.1371/journal.pmed.1002855 PMC6638737

[CIT0014] Lehtimäki K, J Rasmussen V, Kukkonen J, Salomonsson B, Arverud E D, Hole R, Fenstadt A M, Brorson S, Jensen S L, Äärimaa V. Low risk of revision after reverse shoulder arthroplasty for acute proximal humeral fractures. JSES Int 2020; 4: 151–5. 32195478 10.1016/j.jses.2019.10.114 PMC7075766

[CIT0015] Lopiz Y, Alcobia-Diaz B, Galan-Olleros M, Garcia-Fernandez C, Picado A L, Marco F. Reverse shoulder arthroplasty versus nonoperative treatment for 3- or 4-part proximal humeral fractures in elderly patients: a prospective randomized controlled trial. J Shoulder Elbow Surg 2019; 28(12): 2259–71. 31500986 10.1016/j.jse.2019.06.024

[CIT0016] Olerud P, Ahrengart L, Ponzer S, Saving J, Tidermark J. Hemiarthroplasty versus nonoperative treatment of displaced 4-part proximal humeral fractures in elderly patients: a randomized controlled trial. J Shoulder Elbow Surg 2011a; 20: 1025–33. 21783385 10.1016/j.jse.2011.04.016

[CIT0017] Olerud P, Ahrengart L, Ponzer S, Saving J, Tidermark J. Internal fixation versus nonoperative treatment of displaced 3-part proximal humeral fractures in elderly patients: a randomized controlled trial. J Shoulder Elbow Surg 2011b; 20: 747–55. 21435907 10.1016/j.jse.2010.12.018

[CIT0018] Petrigliano F A, Bezrukov N, Gamradt S C, SooHoo N F. Factors predicting complication and reoperation rates following surgical fixation of proximal humeral fractures. J Bone Joint Surg Am 2014; 96: 1544–51. 25232078 10.2106/JBJS.M.01039

[CIT0019] Rajaee S S, Yalamanchili D, Noori N, Debbi E, Mirocha J, Lin C A, Moon C N. Increasing use of reverse total shoulder arthroplasty for proximal humerus fractures in elderly patients. Orthopedics 2017; 40: e982–e89. 28968474 10.3928/01477447-20170925-01

[CIT0020] Rangan A, Handoll H, Brealey S, Jefferson L, Keding A, Martin B C, Goodchild L, Chuang L H, Hewitt C, Torgerson D. Surgical vs nonsurgical treatment of adults with displaced fractures of the proximal humerus: the PROFHER randomized clinical trial. JAMA 2015; 313: 1037–47. 25756440 10.1001/jama.2015.1629

[CIT0021] Rosas S, Law T Y, Kurowicki J, Formaini N, Kalandiak S P, Levy J C. Trends in surgical management of proximal humeral fractures in the Medicare population: a nationwide study of records from 2009 to 2012. J Shoulder Elbow Surg 2016; 25: 608–13. 26475637 10.1016/j.jse.2015.08.011

[CIT0022] Spross C, Meester J, Mazzucchelli R A, Puskás G J, Zdravkovic V, Jost B. Evidence-based algorithm to treat patients with proximal humerus fractures: a prospective study with early clinical and overall performance results. J Shoulder Elbow Surg 2019; 28: 1022–32. 31003888 10.1016/j.jse.2019.02.015

[CIT0023] Sumrein B O, Huttunen T T, Launonen A P, Berg H E, Fellander-Tsai L, Mattila V M. Proximal humeral fractures in Sweden: a registry-based study. Osteoporos Int 2017; 28: 901–07. 27787593 10.1007/s00198-016-3808-z

[CIT0024] van der Merwe M, Boyle M J, Frampton C M A, Ball C M. Reverse shoulder arthroplasty compared with hemiarthroplasty in the treatment of acute proximal humeral fractures. J Shoulder Elbow Surg 2017; 26: 1539–45. 28412103 10.1016/j.jse.2017.02.005

[CIT0025] Wennergren D, Ekholm C, Sandelin A, Möller M. The Swedish fracture register: 103,000 fractures registered. BMC Musculoskeletal Disorders 2015; 16: 338. 26546157 10.1186/s12891-015-0795-8 PMC4636773

[CIT0026] Wennergren D, Stjernstrom S, Moller M, Sundfeldt M, Ekholm C. Validity of humerus fracture classification in the Swedish fracture register. BMC Musculoskelet Disord 2017; 18: 251. 28601085 10.1186/s12891-017-1612-3 PMC5466790

[CIT0027] Wennergren D, Bergdahl C, Selse A, Ekelund J, Sundfeldt M, Möller M. Treatment and re-operation rates in one thousand and three hundred tibial fractures from the Swedish Fracture Register. Eur J Orthop Surg Traumatol 2021; 31(1): 143–54. 32743684 10.1007/s00590-020-02751-x PMC7815548

[CIT0028] Zhu Y, Lu Y, Shen J, Zhang J, Jiang C. Locking intramedullary nails and locking plates in the treatment of two-part proximal humeral surgical neck fractures: a prospective randomized trial with a minimum of three years of follow-up. J Bone Joint Surg Am 2011; 93: 159–68. 21248213 10.2106/JBJS.J.00155

